# Intermedin Alleviates Vascular Calcification in CKD through Sirtuin 3-Mediated Inhibition of Mitochondrial Oxidative Stress

**DOI:** 10.3390/ph15101224

**Published:** 2022-10-02

**Authors:** Shi-Meng Liu, Ya-Rong Zhang, Yao Chen, Deng-Ren Ji, Jie Zhao, Su Fu, Mo-Zhi Jia, Yan-Rong Yu, Chao-Shu Tang, Wei Huang, Ye-Bo Zhou, Yong-Fen Qi

**Affiliations:** 1 Laboratory of Cardiovascular Bioactive Molecule, School of Basic Medical Sciences, Peking University, Beijing 100083, China; 2 Key Laboratory of Molecular Cardiovascular Science, Ministry of Education, Peking University Health Science Center, Beijing 100083, China; 3 Department of Pathogen Biology, School of Basic Medical Sciences, Peking University, Beijing 100083, China; 4 Department of Physiology, School of Basic Medical Sciences, Chongqing Medical University, Chongqing 400016, China; 5 Department of Neurobiology, School of Basic Medical Sciences, Peking University, Beijing 100083, China; 6 Department of Physiology and Pathophysiology, School of Basic Medical Sciences, Peking University, Beijing 100083, China; 7 Institute of Cardiovascular Sciences and Key Laboratory of Molecular Cardiovascular Sciences, School of Basic Medical Sciences, Ministry of Education, Peking University Health Science Center, Beijing 100083, China; 8 Department of Physiology, Nanjing Medical University, Nanjing 211166, China

**Keywords:** intermedin, vascular calcification, sirtuin 3, mitochondrial oxidative stress, chronic kidney disease

## Abstract

Vascular calcification (VC) is a common pathophysiological process of chronic kidney disease (CKD). Sirtuin 3 (Sirt3), a major NAD^+^-dependent protein deacetylase predominantly in mitochondria, is involved in the pathogenesis of VC. We previously reported that intermedin (IMD) could protect against VC. In this study, we investigated whether IMD attenuates VC by Sirt3-mediated inhibition of mitochondrial oxidative stress. A rat VC with CKD model was induced by the 5/6 nephrectomy plus vitamin D_3_. Vascular smooth muscle cell (VSMC) calcification was induced by CaCl_2_ and β-glycerophosphate. IMD_1-53_ treatment attenuated VC in vitro and in vivo, rescued the depressed mitochondrial membrane potential (MMP) level and decreased mitochondrial ROS levels in calcified VSMCs. IMD_1-53_ treatment recovered the reduced protein level of Sirt3 in calcified rat aortas and VSMCs. Inhibition of VSMC calcification by IMD_1-53_ disappeared when the cells were Sirt3 absent or pretreated with the Sirt3 inhibitor 3-TYP. Furthermore, 3-TYP pretreatment blocked IMD_1-53_-mediated restoration of the MMP level and inhibition of mitochondrial oxidative stress in calcified VSMCs. The attenuation of VSMC calcification by IMD_1-53_ through upregulation of Sirt3 might be achieved through activation of the IMD receptor and post-receptor signaling pathway AMPK, as indicated by pretreatment with an IMD receptor antagonist or AMPK inhibitor blocking the inhibition of VSMC calcification and upregulation of Sirt3 by IMD_1-53_. AMPK inhibitor treatment reversed the effects of IMD_1-53_ on restoring the MMP level and inhibiting mitochondrial oxidative stress in calcified VSMCs. In conclusion, IMD attenuates VC by improving mitochondrial function and inhibiting mitochondrial oxidative stress through upregulating Sirt3.

## 1. Introduction

Vascular calcification (VC) refers to deposits of calcium and phosphate crystals in the form of hydroxyapatite precipitating in the vessel wall, which is a common pathophysiological process of chronic kidney disease (CKD), atherosclerosis, hypertension, diabetes, and aging [1,2]. Clinical studies have shown that the occurrence of aortic and coronary calcification in high-risk populations significantly increases all-cause and cardiovascular mortality [3]. However, the mechanism of VC is still not fully understood, and there are still no effective therapies for VC.

At present, VC is considered a highly regulated process that involves the phenotypic transformation of vascular smooth muscle cells (VSMCs) into osteoblast-like cells. During this process, VSMCs lose their contractile markers, such as alpha-smooth muscle actin (αSMA) and smooth muscle-22 alpha (SM22α), but express osteoblast markers, including runt-related transcription factor 2 (Runx2) and bone morphogenetic protein 2 (BMP2) [4,5,6]. Numerous factors, such as imbalanced phosphate/calcium metabolism, uremic toxins, inflammatory cytokines, and oxidative stress, have been identified as contributors to VSMC osteogenic phenotype transformation [7]. Mitochondria, as cellular centers of energy metabolism, maintain the balance between ROS production and antioxidative effects. However, pathological conditions, including CKD, lead to mitochondrial dysfunction that disrupts this balance, leading to mitochondrial-derived oxidative stress and promoting VSMC osteogenic phenotype transformation [8,9]. In contrast to mitochondrial dysfunction, Sirtuin 3 (Sirt3) plays an important role in mitochondrial homeostasis and has been demonstrated to attenuate VC in CKD by maintaining mitochondrial homeostasis [10,11].

Sirt3 is the major NAD+-dependent protein deacetylase predominantly in mitochondria [12]. Impaired mitochondrial function causes hyperacetylation of multiple mitochondrial proteins, which affects protein function. Sirt3 regulates the proper acetylation level of mitochondrial proteins through deacetylation, thus maintaining mitochondrial protein function and ultimately contributing to mitochondrial homeostasis [12]. It has been reported that Sirt3 deacetylates the mitochondrial antioxidant SOD2 to maintain the enzymatic activity of SOD2, thereby preventing overproduction of mitochondria-derived ROS [13]. Previous studies have found that CKD leads to downregulation of Sirt3 in calcified VSMC while restoring Sirt3 expression could alleviate VC in CKD by improving mitochondrial function and inhibiting mitochondrial ROS overproduction [10,11]. Therefore, increasing the expression of Sirt3 may have therapeutic value to alleviate mitochondrial oxidative stress and VC in CKD.

Many studies have shown that endogenous paracrine/autocrine factors are involved in VC [14,15]. Intermedin (IMD) is a member of the calcitonin gene-related peptide (CGRP) superfamily discovered in 2004 [16,17]. IMD_1-53_ is formed by cleavage between arginine residues 93 and 94 of human prepro-IMD. IMD_1-53_ is the longest IMD active fragment and exerts the strongest biological effects [18,19]. IMD exerts its biological effects by non-selectively binding to calcitonin receptor-like receptor (CRLR) and receptor activity modifying protein (RAMP) 1, 2 and 3 [19]. Our previous studies found that IMD alleviated VC by upregulating the anti-aging factor Klotho and the calcification inhibitor matrix γ-carboxyglutamic acid (Gla) protein [14,15]. Importantly, we reported that another Sirtuin family member Sirt1, localized in the cytoplasm and nucleus, is regulated by IMD and involved in IMD alleviating aging-related VC [12]. Considering that Sirt3 contributes to the alleviation of VC and may be regulated by similar upstream signaling pathways as Sirt1 [20,21], we hypothesize that IMD inhibits mitochondrial oxidative stress by regulating Sirt3 and ultimately attenuates VC in CKD.

In this study, we investigated whether IMD alleviates VC in CKD by Sirt3-mediated inhibition of mitochondrial oxidative stress. IMD protected against VC in CKD and increased the protein level of Sirt3 in calcified aortas in vitro and in vivo. Furthermore, IMD-induced Sirt3 upregulation improved mitochondrial function and inhibited mitochondrial oxidative stress in calcified VSMCs. Mechanistically, IMD may upregulate Sirt3 through its receptor complexes and AMPK.

## 2. Materials and Methods

### 2.1. Materials

Synthetic human IMD_1-53_ (Cat. No 010-48) and IMD_17-47_ (Cat. No 010-57) were from Phoenix Pharmaceuticals (Belmont, CA, USA). Alzet mini-osmotic pumps (Alzet model 2004) were from DURECT Corp. (Cupertino, CA, USA). β-glycerophosphate (Cat. No G9422), cholecalciferol (Cat. No C9774), dihydroethidium (DHE) (Cat. No D7008), elastase I (Cat. No E1250), gelatin (Cat. No G7041), hexadecylpyridinium chloride (Cat. No C9002), Compound C (Cat. No 171260), H89 (Cat. No 371962), and LY294002 (Cat. No 440202) were purchased from Sigma. (St. Louis, MO, USA). Type II collagenase (Cat. No LS004176) was purchased from Worthington (Lakewood, NJ, USA). 7C, 3-(1H-1, 2,3-triazol-4-yl) pyridine (3-TYP) (Cat. No S8628) was purchased from Selleck Chemicals (Houston, TX, USA). 4′,6-diamidino-2-phenylindole (DAPI) (Cat. No D3571), MitoSOX^TM^ Red (Cat. No M36008) and MitoTracker^TM^ Green (Cat. No M7541) were purchased from Thermo Fisher Scientific (Waltham, MA, USA). The mitochondrial membrane potential assay kit (Cat. No C2006) and MnSOD activity assay kit (Cat. No S0103) were purchased from Beyotime Biotechnology (Shanghai, China). The primary antibody against β-actin (sc-47778) and all horseradish peroxidase-conjugated secondary antibodies (sc-2357, sc-2354 and sc-2005) were purchased from Santa Cruz Biotechnology (Santa Cruz, CA, USA). Primary antibodies against αSMA (ab5694), SM22α (ab10135), Runx2 (ab23981), BMP2 (ab14933), SOD2 (ab68155) and acetyl-SOD2 (K68) (ab137037) were purchased from Abcam (Cambridge, UK); Sirt3 (5490), AMP-activated protein kinase (AMPK) (2532), phospho-AMPK (T172) (2531), Akt (9272), phosphor-Akt (S473) (4060) were purchased from Cell Signaling Technology (Danvers, MA, USA). Protein kinase A (PKA) (sc28315) and phospho-PKA (T197) (sc32968) were purchased from Santa Cruz Biotechnology (Santa Cruz, CA, USA). All DyLight-labeled secondary antibodies (E032220 and E032330) were purchased from EarthOx (Millbrae, CA, USA). An enhanced chemiluminescence kit (Cat. No P1050) was purchased from Beijing Applygen Technologies (Beijing, China). Other chemicals and reagents were of analytical grade.

### 2.2. Rat VC in the CKD Model

Eight-week-old male Sprague–Dawley (SD) rats were purchased from the Animal Center, Peking University Health Science Center (Beijing, China). All animal care and experimental protocols complied with the Guide for the Care and Use of Laboratory Animals published by the US National Institutes of Health (NIH Publication, 8th Edition, 2011) and were approved by the Animal Care Committee of Peking University Health Science Center. Rats were randomly assigned to 3 groups for treatment (*n* = 6–8 for each group): control, CKD, and CKD + IMD. Rat CKD was prepared by five-sixths nephrectomy as described [1] with minor modification. Pentobarbital sodium (40 mg/mL) was used to anesthetize rats with intraperitoneal injection. Briefly, the right kidney of the rat was removed surgically. One week later, two-thirds left nephrectomy was performed by amputation of both renal poles. Vitamin D_3_ was administered intramuscularly at 1 mg/kg body weight (0.1621 mg/kg body weight in human according to human effective dose (HED) formula [2]) 3 times a week for 12 weeks. Penicillin (200,000 U/kg) was given postoperatively for 3 days. For the CKD + IMD group, IMD_1–53_ was administered subcutaneously (100 ng/kg/h, equal to 1.61 × 10^−5^ mg/kg/h for human according to HED formula [2]) in phosphate buffered saline (PBS) during the last 4 weeks of CKD treatment via an Alzet Mini-osmotic Pump.

Twelve weeks after five-sixths nephrectomy, the rats were sacrificed. Blood was collected for biochemical assays, and the aortas were immediately removed and subjected to morphology studies or immunoblot analysis.

### 2.3. VSMC Culture and VSMC Calcification Model

Rat VSMCs were isolated from the thoracic aortas of SD rats (120–150 g) [3]. Briefly, after partial removal of external connective tissues, the rat thoracic aortas were cut into small pieces (approximately 2–3 mm each), placed in Dulbecco’s modified Eagle’s medium (DMEM) containing 20% fetal bovine serum (FBS), 100 U/mL penicillin and 100 μg/mL streptomycin, and incubated at 37 °C in an incubator containing 95% air and 5% CO_2_. VSMCs migrating from explants were collected and maintained in DMEM containing 10% FBS. VSMCs at passages 5 to 8 were used for all experiments.

Sirt3^-/-^ mice were kindly provided by Prof. Houzao Chen (Peking Union Medical College, Beijing, China). Mouse VSMCs were isolated from 8-week Sirt3^-/-^ mice or WT littermate control mice as described [3]. Briefly, the thoracic aortas were digested with 1 mg/mL type II collagenase at 37 °C for 15 min. The adventitia and endothelium were removed. Aortas were placed in DMEM containing 10% FBS, 100 U/mL penicillin and 100 μg/mL streptomycin overnight in a 37 °C incubator with 5% CO_2_ and then digested in 5 mL of type II collagenase (1 mg/mL) and elastase I (0.25 mg/mL) for 90 min at 37 °C. The cellular digests were centrifuged at 1000 rpm for 3 min, and cells were cultured in DMEM containing 20% FBS in culture dishes coated with gelatin at 37 °C and 5% CO_2_. The cells at passages 4 to 6 were used.

For calcification, confluent VSMCs were incubated in medium containing 2.5 mmol/L Ca^2+^ (0.7 mmol/L CaCl_2_ was added to DMEM containing 1.8 mmol/L CaCl_2_) and 5 mmol/L β-glycerophosphate. The medium was replaced every 2 to 3 days [4].

For IMD_1-53_ treatment, the cells were treated with 10^−7^ mol/L IMD_1-53_ for 30 min, and calcification was induced. For Sirt3 inhibition, 50 μm/L 3-TYP was added to the medium for 30 min before IMD_1-53_ treatment and calcification induction. For IMD receptor antagonization, 10^−6^ mol/L IMD_17-47_ was added to the medium for 30 min before IMD_1-53_ treatment and calcification induction. For inhibition of the signaling pathway, 10 μmol/L phosphatidylinositol 3-kinase (PI3K) inhibitor LY294002, AMPK inhibitor Compound C or PKA inhibitor H89 was added to the medium for 30 min before IMD_1-53_ treatment and calcification induction.

### 2.4. Biochemistry

Blood obtained from abdominal aortas at the end of the experiment was analyzed for levels of calcium, phosphate, blood urea nitrogen (BUN), and creatinine. Plasma calcium, phosphate, BUN, and creatinine levels were determined by an auto chemistry analyzer.

### 2.5. Hematoxylin and Eosin Staining

Segments of rat thoracic aortas were placed in 4% paraformaldehyde for 8 h and then immersed in 20% sucrose solution for storage; aorta samples were dehydrated and embedded in paraffin, cut into 5-µm-thick sections, and then subjected to H&E staining as described previously [3].

### 2.6. Alizarin Red Staining

To examine aortic calcification, slides were dehydrated, rinsed rapidly in distilled water, and placed in alizarin red staining solution (pH 4.2, 1%) at room temperature for 5 min. When the positive red–orange color appeared, the stained tissue was washed again with distilled water to remove any unbound staining. Then, the tissue was photographed [3].

To examine VSMC calcification, cultured VSMCs grown in 12-well plates were washed with PBS 3 times and then fixed in 4% paraformaldehyde for 15 min. The cells were washed with distilled water, exposed to alizarin red staining solution (pH 4.2, 1%) for 30 min, washed again with distilled water and observed by microscopy [3]. To quantify alizarin red staining, 200 μL hexadecylpyridinium chloride solution (100 mmol/L) was added to the wells, and absorbance was measured at 570 nm using hexadecylpyridinium chloride solution as a blank [5].

### 2.7. ROS Analysis

Dihydroethidium (DHE) was used to detect ROS in thoracic aortas and VSMCs. ROS production in the thoracic aorta was measured as previously described [6] with minor modifications. Thoracic aortas were embedded in OCT and snap-frozen. Freshly cut frozen aortic sections (7 μm) were incubated with 5 μmol/L DHE for 30 min at 37 °C. Then, the sections were washed with distilled water and fixed in 4% paraformaldehyde for 15 min. Sections were examined by fluorescence microscopy to reveal the presence of ROS as red fluorescence (585 nm). To measure ROS generation in live VSMCs, cultured VSMCs grown in 12-well plates were washed with PBS 3 times and then incubated with DMEM containing 5 μmol/L DHE at 37 °C in an incubator containing 95% air and 5% CO_2_ for 30 min to load the probe. The cells were washed with PBS 3 times again after DHE loading, and the presence of ROS was observed at 580 nm by a fluorescence microscope.

MitoSOX^TM^ Red was used to detect mitochondrial superoxide in live VSMCs. Cultured VSMCs grown in 12-well plates were washed with PBS 3 times and then incubated with DMEM containing 5 μmol/L MitoSOX^TM^ Red at 37 °C in an incubator containing 95% air and 5% CO_2_ for 10 min to load the probe. The cells were washed with PBS 3 times again, and the presence of mitochondrial superoxide was observed at 580 nm by a fluorescence microscope.

### 2.8. SOD Activity Assay

For the detection of total SOD activity and mitochondrial-specific SOD2 activity, the cell lysate of VSMCs was detected with the MnSOD Assay Kit with WST-8 according to the manufacturer’s protocol. Briefly, for the SOD2 activity assay, the SOD1 inhibitor compound was added to the cell lysate to inhibit residual SOD1 activity, followed by incubation with the WST-8/enzyme working solution at 37 °C for 30 min. For total SOD activity, the cell lysate was incubated with the WST-8/enzyme working solution at 37 °C for 30 min directly. The absorption was measured at a wavelength of 450 nm. When the inhibition rate of WST-8 formazan was 50%, the enzymatic activity of SOD2 was defined as one unit. The protein concentrations were determined using a BCA assay. The results were expressed as units per mg protein (U/mg prot).

### 2.9. Mitochondrial Membrane Potential Measurements

The mitochondrial membrane potential (MMP) level was measured using a MMP assay kit with JC-1 according to the manufacturer’s protocol. Briefly, JC-1 accumulated into J-aggregates in the mitochondrial matrix and showed red fluorescence under conditions of a higher MMP level, whereas the JC-1 monomer showed green fluorescence when the MMP level was lower. Images were captured under a fluorescence microscope. The red and green JC-1 fluorescence densities were analyzed by ImageJ (National Institutes of Health).

### 2.10. Enzyme-Linked Immunosorbent Assay

Blood samples were collected from rats, and plasma was obtained by centrifugation at 3000 rpm for 10 min at 4 °C. The ELISA kit for mouse IMD was used. The cross reactivity of the mouse IMD ELISA kit was 100% with rat IMD, so we detected rat plasma IMD using the mouse IMD ELISA kit. The concentration of IMD was determined according to the instructions of the manufacturer. The minimum detectable concentration of IMD was 0.22 ng/mL. The intra- and inter-assay coefficient of variation were less than 10% and 15%, respectively. The detection range among the kits was 0.22 to 7.2 ng/mL.

### 2.11. Immunofluorescence Staining

Sections of thoracic aortas (7 µm) were freshly embedded in OCT and subjected to immunofluorescence staining. Sections were first fixed in 4% paraformaldehyde for 15 min and permeabilized with 0.1% Triton X-100 for 10 min. Nonspecific binding was reduced by incubating slides in PBS containing 5% bovine serum albumin for 60 min at 37 °C. Sections were incubated with antibody against Sirt3 (1:100) at 4 °C overnight, rinsed with PBS and incubated with secondary antibody. Nuclei were counterstained with DAPI. Images were acquired under a fluorescence microscope.

Cultured VSMCs grown in 12-well plates were washed with PBS 3 times and incubated with DMEM containing 100 nmol/L MitoTracker^TM^ Green FM at 37 °C in an incubator containing 95% air and 5% CO_2_ for 30 min. The cells were washed with PBS 3 times and fixed in 4% paraformaldehyde for 10 min. Then, the cells were incubated with PBS containing 5% bovine serum albumin and 0.1% Triton X-100 for 60 min at room temperature to reduce nonspecific binding and permeabilization. The cells were incubated with antibody against Sirt3 (1:100) at room temperature for 1 h, rinsed with PBS and incubated with fluorescein-labeled secondary antibody. Nuclei were counterstained with DAPI. Images were acquired by a fluorescence microscope.

### 2.12. Western Blot Analysis

Aortic tissues or cell extracts containing equal amounts of total protein were resolved by 10% SDS–PAGE and then transferred to a nitrocellulose membrane. Nonspecific proteins were blocked with 5% nonfat dried milk for 1 h and then incubated with primary antibodies against β-actin (1:3000); αSMA (1:3000), SM22α (1:1000), RUNX2 (1:1000), BMP2 (1:1000); Sirt3 (1:1000); SOD2 (1:3000) and acetyl-SOD2 (1:1000); Nrf2 (1:1000); AMPK (1:1000), p-AMPK (Thr172) (1:1000), Akt (1:1000), p-Akt (Ser473) (1:1000), PKA (1:1000) or p-PKA (Thr197) (1:1000) overnight at 4 °C and then with a secondary antibody (horseradish peroxidase-conjugated anti-rabbit, anti-mouse or anti-goat IgG) for 1 h. The proteins were detected by enhanced chemiluminescence. The protein level was analyzed by using ImageJ and normalized to β-actin expression.

### 2.13. Statistical Analysis

Normal distribution was assessed by the Shapiro-Wilk test. Homogeneity of variances was assessed by the F test or Brown-Forsythe test. All data showed normal distribution and passed equal variance testing. The data are expressed as the mean ± SD. Statistical analysis was performed using GraphPad Prism v7.00 (GraphPad Software Inc., San Diego, CA, USA). Student’s *t* test was used to compare two groups, and one-way ANOVA followed by Tukey’s *post-hoc* test was used for multiple group comparisons. Statistical significance was accepted at *p* < 0.05.

## 3. Results

### 3.1. IMD Attenuated VC in CKD Rats by Improving Mitochondrial Function and Inhibiting Mitochondrial Oxidative Stress

To confirm the role of IMD in VC, we first examined the IMD level in plasma and its receptors in the aortas of CKD rats. Disordered elastic fibers and increased calcium-phosphate deposition were observed in the aortas of CKD rats (Figure S1A,B). The level of IMD in plasma was decreased significantly in CKD rats compared to the control group (Figure S1D). The protein levels of the IMD receptor complexes CRLR, RAMP2 and RAMP3 in the calcified aortas of CKD rats were increased significantly compared with those of controls. The protein level of RAMP1 was decreased compared with that in the control group (Figure S1E–I). These data was consistent with our previous report [4].

**Figure 1:**
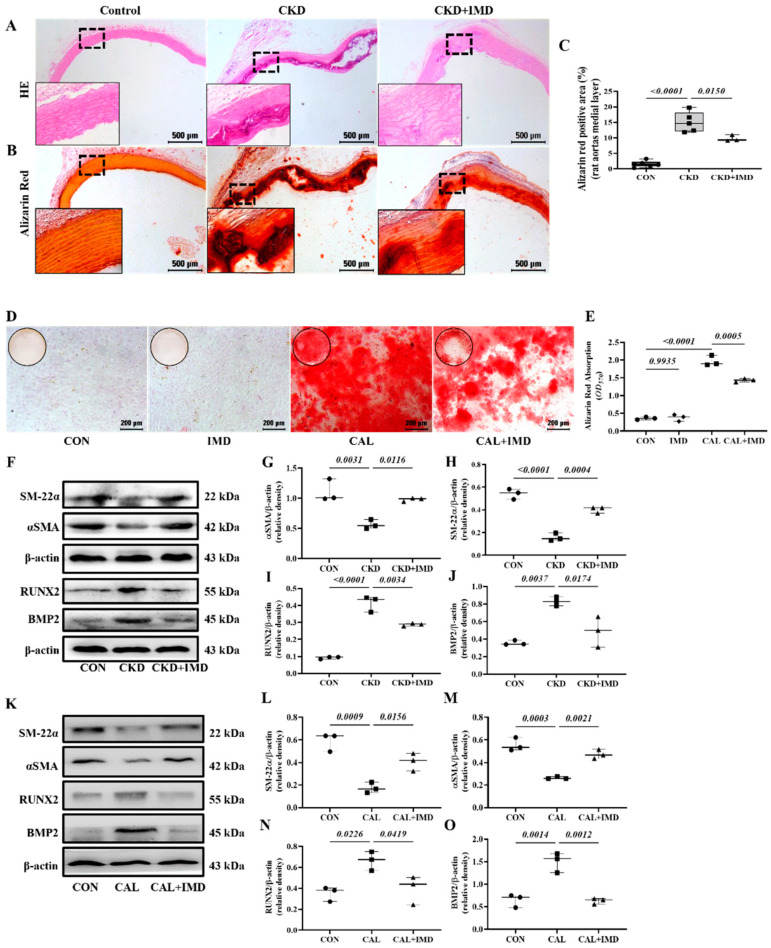
IMD alleviated vascular calcification in CKD rats and calcification of VSMCs. (**A**) H&E staining (Scale bar = 500 μm) and (**B**) Alizarin red staining for calcium deposition (positive staining: red) (Scale bar = 500 μm) in rat aortas. (**C**) Quantification of alizarin red-positive staining (*n* = 3–6). (**D**) Alizarin red staining (positive staining: red) (Scale bar = 200 μm) of calcified rat VSMCs and (**E**) quantification (*n* = 3). (**F**) Western blot analysis of the protein levels of smooth muscle 22 alpha (SM22α), alpha smooth muscle actin (αSMA), bone morphogenetic protein 2 (BMP2) and runt-related transcription factor 2 (RUNX2) in rat aortas and (**G**–**J**) quantification (*n* = 3). (**K**) Western blot analysis of the protein levels of SM-22α, αSMA, BMP2 and RUNX2 in rat VSMCs and (**L**–**O**) quantification (*n* = 3). CON (circles): control rats or VSMCs, CKD (squares in (**C**,**G**–**J**)): CKD vascular calcification rats, CKD + IMD (triangles in (**C**,**G**–**J**)): CKD vascular calcification rats with IMD_1-53_ treatment, IMD (diamonds in (**E**)): control VSMCs with IMD_1-53_ treatment, CAL (squares in (**E**,**L**–**O**)): calcification VSMCs, CAL + IMD (triangles in (**E**,**L**–**O**)): calcification VSMCs with IMD_1-53_ treatment. Data are mean ± SD.

Exogenous IMD_1-53_ significantly improved renal function, with decreased plasma Cr and BUN levels and decreased plasma calcium and phosphorus levels in CKD rats (Figure S2). Notably, IMD_1-53_ treatment improved vascular structure and reduced calcium deposition in the aortas of CKD rats (Figure 1A–C). In vitro, VSMCs (validated by immunofluorescence staining showing positive expression of the VSMC lineage marker αSMA (Figure S3A)) cultured in calcified medium showed significantly increased calcium deposition compared to the cells cultured in conventional medium, while administration of IMD_1-53_ significantly reduced calcium deposition from the calcified cells (Figure 1D,E). Phenotypic transition of VSMCs plays a pivotal role in VC [7]. We confirmed that IMD treatment inhibited the osteoblast-like phenotypic transition of VSMCs, as evidenced by increased protein levels of the VSMC lineage markers SM22α and αSMA and decreased protein levels of the osteogenic markers Runx2 and BMP2 in calcified aortas of CKD rats (Figure 1F–J). Furthermore, we confirmed that IMD_1-53_ treatment inhibited VSMC transformation into osteoblast-like cell in vitro (Figure 1K–O).

**Figure 2:**
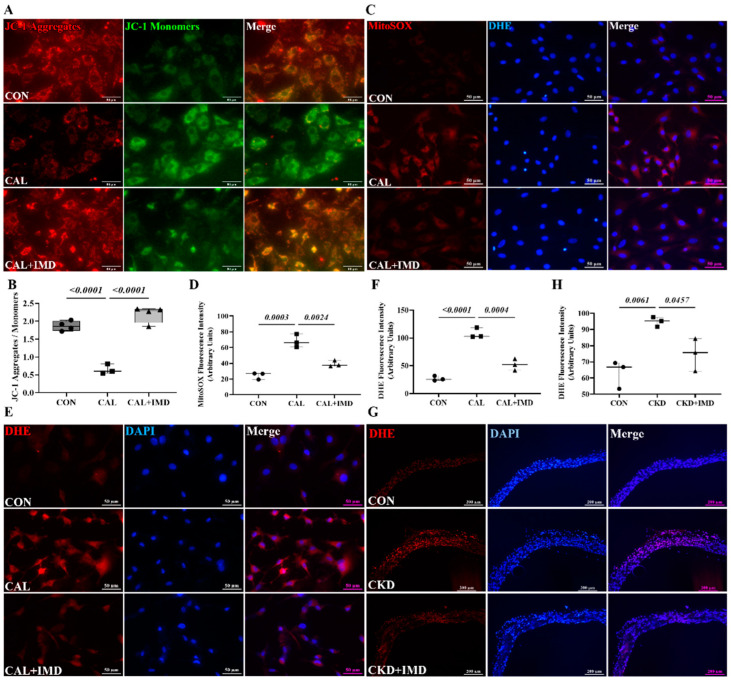

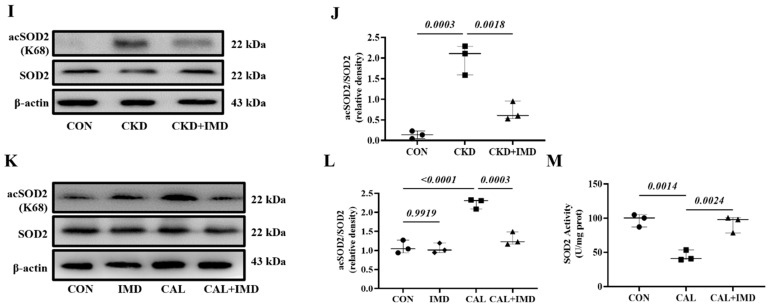
IMD inhibited mitochondrial oxidative stress in calcified VSMCs. (**A**) JC-1 probing mitochondrial membrane potential in rat VSMCs (red: JC-1 aggregates, green: JC-1 monomers). Merged images are shown (Scale bar = 50 μm). (**B**) Ratio of average fluorescence intensity of JC-1 aggregates to JC-1 monomers (*n* = 3). (**C**) MitoSOX Red probing mitochondrial ROS in rat VSMCs (positive staining: red). Nuclei were stained with DAPI (blue). Merged images are shown (Scale bar = 50 μm). (**D**) Average fluorescence intensity of MitoSOX in rat VSMCs (*n* = 3). (**E**) DHE probing ROS in rat VSMCs (positive staining: red). Nuclei were stained with DAPI (blue). Merged images are shown (Scale bar = 50 μm). (**F**) Average fluorescence intensity of DHE in rat VSMCs (*n* = 3). (**G**) DHE probing ROS in rat aortas (positive staining: red). Nuclei were stained with DAPI (blue). Merged images are shown (Scale bar = 200 μm). (**H**) Average fluorescence intensity of DHE in rat aortas (*n* = 3). Western blot analysis of protein levels of acetylation-SOD2 (K68) and SOD2 in (**I**) rat aortas and (**K**) rat VSMCs, and (**J**,**L**) quantification of acetylation-SOD2 to SOD2 ratio (*n* = 3). (**M**) SOD2 activity in rat VSMCs (*n* = 3). CON (circles): control rats or VSMCs, CKD (squares in (**H**,**J**)): CKD vascular calcification rats, CKD + IMD (triangles in (**H**,**J**)): CKD vascular calcification rats with IMD_1-53_ treatment, IMD (diamonds in (**L**)): control VSMCs with IMD_1-53_ treatment, CAL (squares in (**B**,**D**,**E**,**L**,**M**)): calcification VSMCs, CAL + IMD (triangles in (**B**,**D**,**E**,**L**,**M**)): calcification VSMCs with IMD_1-53_ treatment. Data are mean ± SD.

Given the importance of mitochondrial homeostasis in VSMC phenotypic transition and VC [8], we examined the effects of IMD on mitochondrial function in calcified VSMCs. As MMP is a well-known indicator of mitochondrial function, the MMP level of VSMCs was measured. The MMP level was decreased in calcified VSMCs compared to controls, and this decrease was reversed by IMD_1-53_ treatment (Figure 2A,B). Correspondingly, the levels of mitochondrial ROS were significantly increased in calcified cells and was reduced by IMD_1-53_ treatment (Figure 2C,D). Furthermore, elevated ROS levels were observed both in calcified aortas of CKD rats and calcified VSMCs, as indicated by enhanced DHE fluorescence intensity, and treatment with IMD reversed these changes (Figure 2E–H). These results indicated that IMD improved mitochondrial function and inhibited mitochondrial oxidative stress.

SOD2 plays a crucial role in oxidative resistance against mitochondrial superoxide [9]. SOD2 activity is mainly regulated by acetylation and is closely related to mitochondrial function [10,11]. Therefore, we further confirmed that IMD improved mitochondrial function by assaying the enzymatic activity and acetylation level of SOD2. IMD_1-53_ reduced the acetylation level of SOD2 in calcified aortas of CKD rats and calcified VSMCs and consequently restored the enzymatic activity of SOD2 (Figure 2I–M). Collectively, these results suggest that IMD attenuated VC in CKD by improving mitochondrial function and inhibiting mitochondrial oxidative stress.

### 3.2. IMD Improved Mitochondrial Function and Inhibited Mitochondrial Oxidative Stress by Upregulating Sirt3

**Figure 3:**
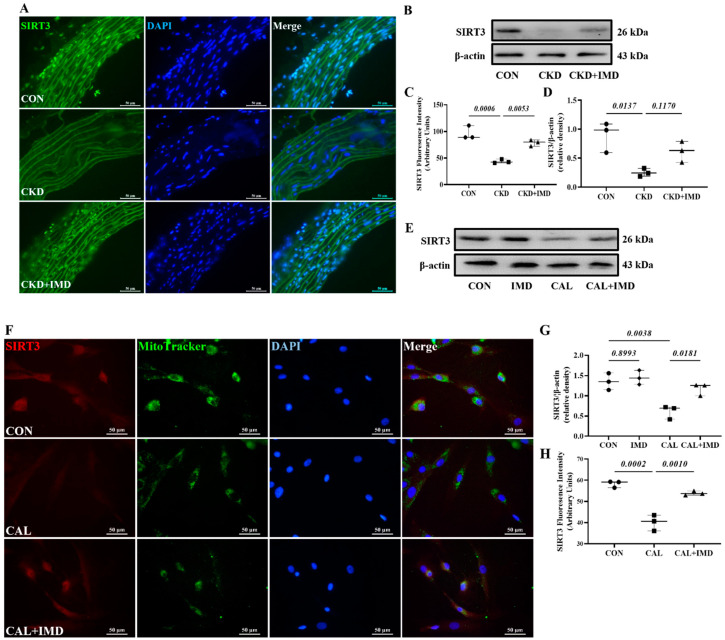
IMD upregulated Sirt3 in the calcified aortas of CKD rats and calcified VSMCs. (**A**) Immunofluorescence staining for Sirt3 (green) in rat aortas. Nuclei were stained with DAPI (blue). Merged images are shown (Scale bar = 50 μm). (**C**) Average fluorescence intensity of Sirt3 in rat aortas (*n* = 3). (**B**) Western blot analysis of protein levels of Sirt3 in rat aortas and (**D**) quantification (*n* = 3). (**E**) Western blot analysis of protein levels of Sirt3 in rat VSMCs and (**G**) quantification (*n* = 3). (**F**) Immunofluorescence staining for Sirt3 (green) in rat VSMCs. Mitochondria were stained with MitoTracker (green). Nuclei were stained with DAPI (blue). Merged images are shown (Scale bar = 50 μm). (**H**) Average fluorescence intensity of Sirt3 in VSMCs (*n* = 3). CON (circles): control rats or VSMCs, CKD (squares in (**C**,**D**): CKD vascular calcification rats, CKD + IMD (triangles in (**C**,**D**)): CKD vascular calcification rats with IMD_1-53_ treatment, IMD (diamonds in (**G**)): control VSMCs with IMD_1-53_ treatment, CAL (squares in (**G**,**H**)): calcification VSMCs, CAL + IMD (triangles in (**G**,**H**)): calcification VSMCs with IMD_1-53_ treatment. Data are mean ± SD.

We further explored how IMD improved mitochondrial function in calcified VSMCs. First, we observed a decrease in the protein level of Sirt3 in both the calcified aortas of CKD rats (Figure 3A–D) and calcified VSMCs (Figure 3E–H). This result is consistent with literature reports [12]. Furthermore, the decreased protein level of Sirt3 was restored by IMD_1-53_ treatment both in vivo (Figure 3A–D) and in vitro (Figure 3E–H), suggesting that IMD could upregulate Sirt3.

**Figure 4:**
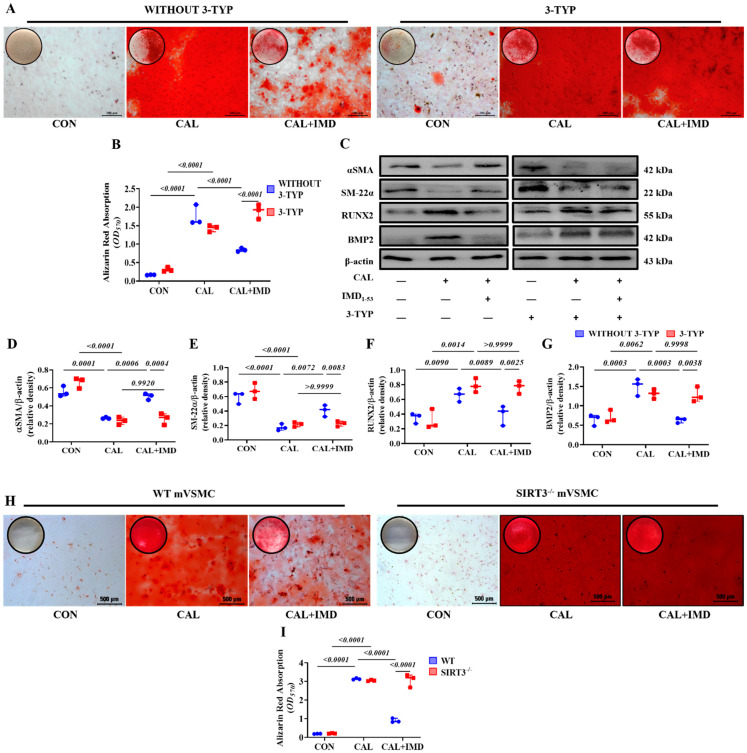
Sirt3 mediated IMD inhibition of vascular calcification. Alizarin red staining (positive staining: red) of (**A**) rat VSMCs (Scale bar = 100 μm) and (**H**) mouse VSMCs (Scale bar = 500 μm) and (**B**,**I**) quantification (*n* = 3). (**C**) Western blot analysis of the protein levels of SM22α, αSMA, BMP2 and RUNX2 in rat VSMCs and (**D**–**G**) quantification (*n* = 3). CON: control VSMCs, CAL: calcification VSMCs, CAL + IMD: calcification VSMCs with IMD_1-53_ treatment. 3-TYP (blue circles in (**B**,**D**–**G**)): VSMCs with 3-TYP pretreatment, WITHOUT 3-TYP (red squares in (**B**,**D**–**G**)): VSMCs without 3-TYP pretreatment. WT (blue circles in (**I**)): VSMCs from wild-type mice, SIRT3^-/-^ (red squares in (**I**)): VSMCs from Sirt3-knockout mice. Data are mean ± SD.

To clarify the role of Sirt3 in IMD attenuating VC, 3-TYP, a selective Sirt3 inhibitor [13], was used. 3-TYP efficiently inhibited Sirt3 enzymatic activity, as indicated by the increased level of SOD2 acetylation in 3-TYP-treated VSMCs (Figure S4). We found that 3-TYP pretreatment significantly blocked the effect of IMD_1-53_ on alleviating VSMC calcification (Figure 4A,B). Moreover, 3-TYP pretreatment abolished the inhibitory effect of IMD_1-53_ on VSMC osteogenic transdifferentiation (Figure 4C–G).

VSMCs from Sirt3-knockout mice (Sirt3^-/-^ mVSMC) and littermate WT mice (WT mVSMC) were obtained to confirm this finding. Sirt3^-/-^ mVSMCs lacked Sirt3 expression but positively expressed the VSMC lineage marker αSMA (Figure S3B). IMD_1-53_ treatment significantly reduced calcium deposition in calcified WT mVSMCs but had no effect on Sirt3^-/-^ mVSMCs (Figure 4H,I). These findings suggest that SIRT3 is required for the inhibitory effect of IMD_1-53_ on VMSC calcification.

**Figure 5:**
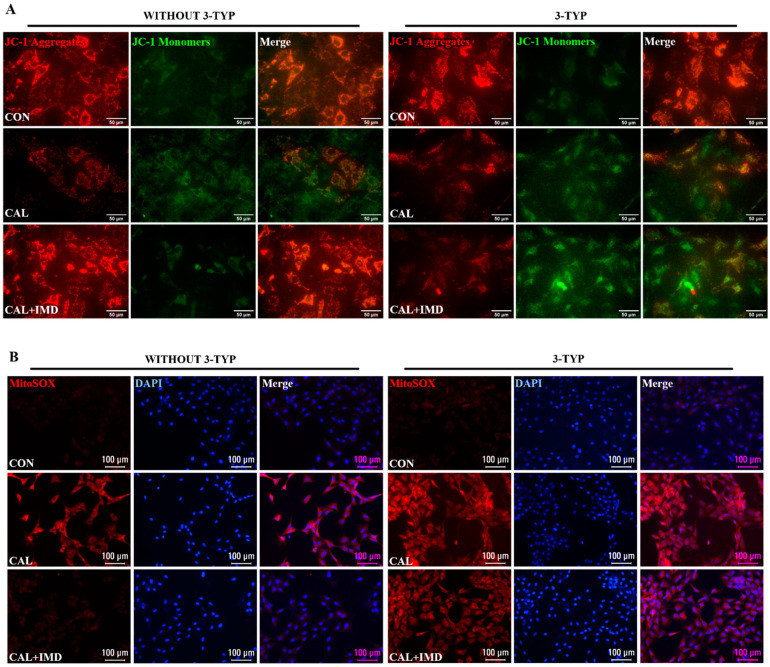

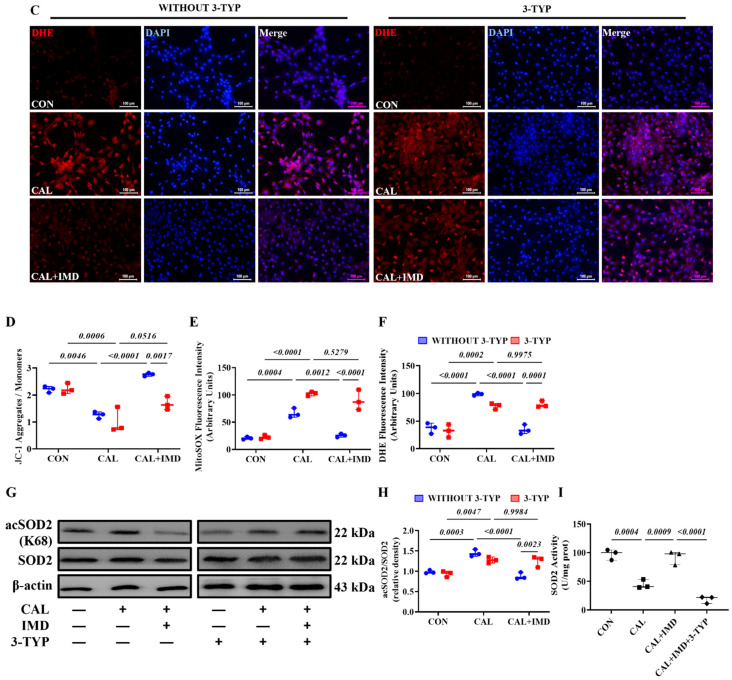
The Sirt3 inhibitor 3-TYP blocked IMD improving mitochondrial function of calcified VSMCs. (**A**) JC-1 probing mitochondrial membrane potential in rat VSMCs (red: JC-1 aggregates, green: JC-1 monomers). Merged images are shown (Scale bar = 50 μm). (**B**) MitoSOX Red probing mitochondrial ROS in rat VSMCs (positive staining: red). Nuclei were stained with DAPI (blue). Merged images are shown (Scale bar = 100 μm). (**C**) DHE probing intracellular ROS in rat VSMCs (positive staining: red). Nuclei were stained with DAPI (blue). Merged images are shown (Scale bar = 100 μm). (**D**) Ratio of the average fluorescence intensity of JC-1 aggregates to JC-1 monomers (*n* = 3). (**E**) Average fluorescence intensity of MitoSOX in rat VSMCs (*n* = 3). (**F**) Average fluorescence intensity of DHE in rat VSMCs (*n* = 3). (**G**) Western blot analysis of the protein levels of acetylation-SOD2 (K68) and SOD2 in rat VSMCs and (**H**) quantification of the acetylation-SOD2 to SOD2 ratio (*n* = 3). (**I**) SOD2 activity in rat VSMCs (*n* = 3). CON (circles in (**I**)): control VSMCs, CAL: (squares in (**I**)) calcification VSMCs, CAL + IMD (triangles in (**I**)): calcification VSMCs with IMD_1-53_ treatment, CAL + IMD + 3-TYP (diamonds in (**I**)): calcification VSMCs with 3-TYP pretreatment and IMD_1-53_ treatment. 3-TYP (blue circles in (**D**–**F**) and (**H**): VSMCs with 3-TYP pretreatment, WITHOUT 3-TYP (red squares in (**D**–**F**) and (**H**)): VSMCs without 3-TYP pretreatment. Data are mean ± SD.

We further used 3-TYP to investigate whether Sirt3 is involved in IMD improving mitochondrial function and inhibiting mitochondrial oxidative stress. 3-TYP pretreatment blocked the effects of IMD on elevating MMP level in calcified VSMCs (Figure 5A,D) and subsequently abolished the inhibitory effects of IMD_1-53_ on mitochondrial and intracellular ROS accumulation in calcified VSMCs (Figure 5B–F). Correspondingly, there was no significant reduction of the SOD2 acetylation level or recovery of SOD2 enzymatic activity in 3-TYP-pretreated calcified VSMCs with IMD_1-53_ administration (Figure 5G–H). Taken together, these results suggest that IMD improves mitochondrial function and inhibits mitochondria-derived oxidative stress in calcified VSMCs by upregulating Sirt3.

### 3.3. IMD Upregulated Sirt3 by Activating Its Receptor and the AMPK Pathway

**Figure 6:**
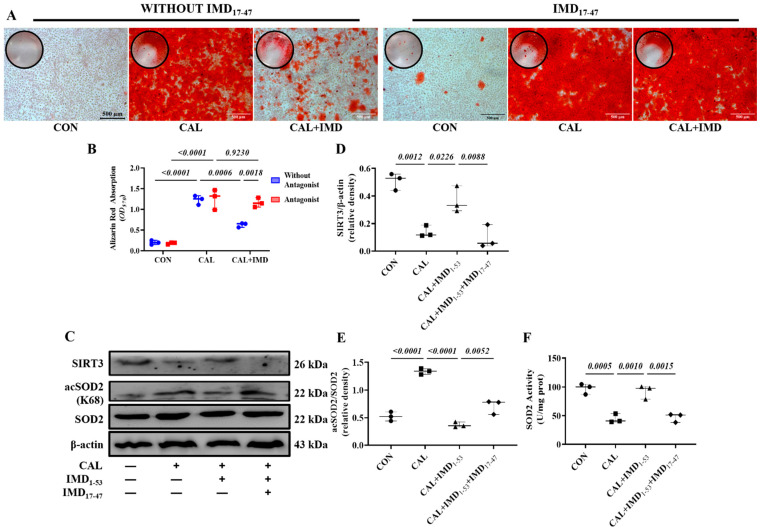
IMD upregulated Sirt3 via its receptor complex. (**A**) Alizarin red staining (positive staining: red) of rat VSMCs (Scale bar = 500 μm), and (**B**) quantification (*n* = 3). (**C**) Western blot analysis of protein levels of Sirt3, acetylation-SOD2 (K68) and SOD2 in rat VSMCs and quantification of (**D**) Sirt3 and (**E**) acetylation-SOD2 to SOD2 ratio (*n* = 3). (**F**) SOD2 activity in rat VSMCs (*n* = 3). CON (circles in (**D**–**F**)): control VSMCs, CAL (squares in (**D**–**F**)): calcification VSMCs, CAL + IMD_1-53_ (triangles in (**D**–**F**)): calcification VSMCs with IMD_1-53_ treatment, CAL + IMD_1-53_ + IMD_17-47_ (diamonds in (**D**–**F**)): calcification VSMCs with IMD_17-47_ pretreatment and IMD_1-53_ treatment. Without Antagonist (blue circles in (**B**)): VSMCs without IMD_17-47_ pretreatment, Antagonist (red squares in (**B**): VSMCs with IMD_17-47_ pretreatment. Data are mean ± SD.

IMD exerts biological effects through its receptor complex CRLR/RAMPs and the post-receptor signaling pathway [3,14]. First, to elucidate whether IMD regulates Sirt3 via CRLR/RAMPs, we used IMD_17–47_, an IMD receptor antagonist. IMD_17-47_ pretreatment completely blocked the effect of IMD_1-53_ on alleviating VSMC calcification (Figure 6A,B). As expected, IMD_17-47_ preincubation blocked the effect of IMD_1-53_ on upregulating Sirt3 in calcified VSMCs (Figure 6C,D). IMD_17-47_ pretreatment abolished the effects of IMD_1-53_ on alleviating SOD2 acetylation and increasing SOD2 activity through upregulation of Sirt3 in calcified cells (Figure 6C,E,F). These results suggested that IMD upregulated Sirt3 through its receptor complex.

**Figure 7:**
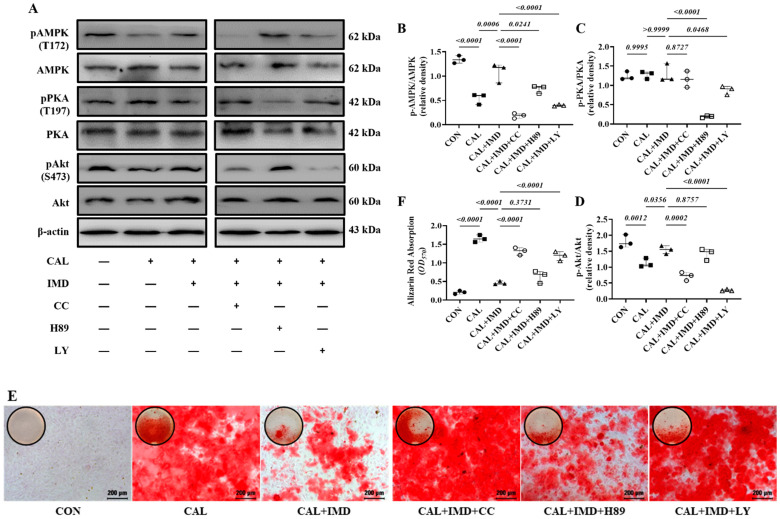

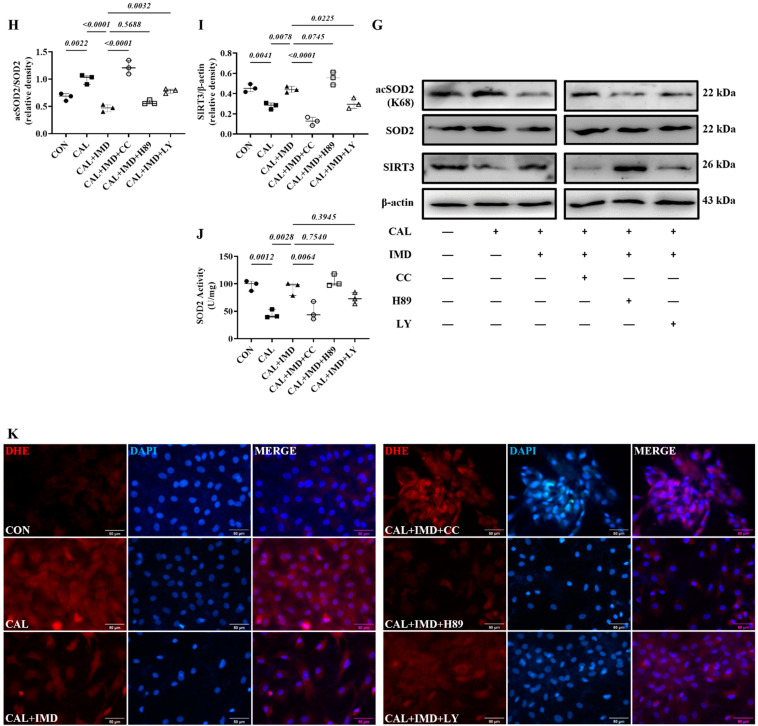

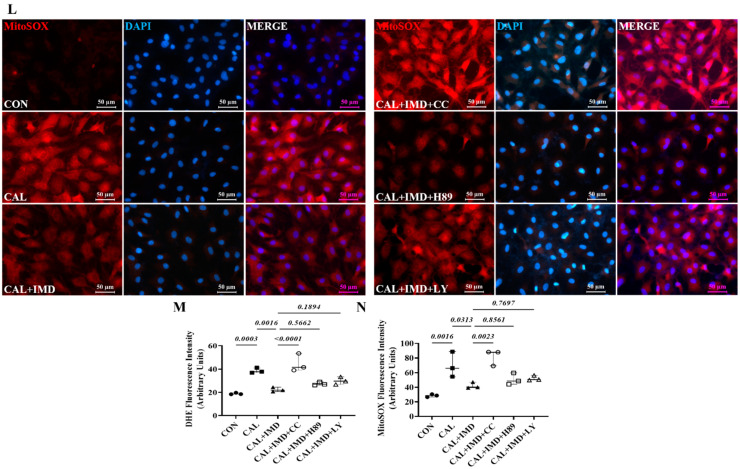
IMD upregulated Sirt3 via the AMPK signaling pathway. (**A**) Western blot analysis of the protein levels of phosphorylated AMP-activated protein kinase (p-AMPK) (T172), AMPK, phosphorylated protein kinase A (p-PKA) (T197), PKA, phosphorylated protein kinase B (p-Akt) (S473), and Akt in rat VSMCs preincubated with or without the phosphatidylinositol 3-kinase (PI3K) inhibitor LY294002, the AMPK inhibitor Compound C or the PKA inhibitor H89 (all 10 μmol/L) before IMD_1–53_ administration and calcification induction and quantification of (**B**) p-AMPK/AMPK, (**C**) p-PKA/PKA, and (**D**) p-Akt/Akt (*n* = 3). (**E**) Alizarin red staining (Scale bar = 200 μm), and (**F**) quantification (*n* = 3). (**G**) Western blot analysis of protein levels of Sirt3, acetylation-SOD2 (K68) and SOD2 in rat VSMCs and quantification of (**H**) acetylation-SOD2 to SOD2 ratio and (**I**) Sirt3 (*n* = 3). (**J**) SOD2 activity in rat VSMCs (*n* = 3). (**K**) DHE probing ROS in rat VSMCs (positive staining: red). Nuclei were stained with DAPI (blue). Merged images are shown (Scale bar = 100 μm). (**L**) MitoSOX Red probing mitochondrial-derived ROS in rat VSMCs (positive staining: red). Nuclei were stained with DAPI (blue). Merged images are shown (Scale bar = 100 μm). (**M**,**N**) Average fluorescence intensity of (**M**) DHE and (**N**) MitoSOX in rat VSMCs (*n* = 3). CON (solid circles): control VSMCs, CAL (solid squares): calcification VSMCs, CAL + IMD (solid triangles): calcification VSMCs with IMD_1-53_ treatment, CAL + IMD + CC (open circles): calcification VSMCs with Compound C pretreatment and IMD_1-53_ treatment, CAL + IMD + H89 (open squares): calcification VSMCs with H89 pretreatment and IMD_1-53_ treatment, CAL + IMD + LY (open triangles): calcification VSMCs with LY294002 pretreatment and IMD_1-53_ treatment Data are mean ± SD.

Post-receptor signaling pathways of IMD include cAMP/PKA, PI3K/Akt, and AMPK [3,15]. To investigate the signaling pathway by which IMD regulates Sirt3, we used the AMPK inhibitor Compound C [16], the PKA inhibitor H89 [17], and the PI3K inhibitor LY294002 [18]. Pretreatment with Compound C or LY294002 blocked the upregulatory effect of IMD_1-53_ on Sirt3 protein levels and, consistently, blocked Sirt3-mediated SOD2 deacetylation. However, only pretreatment with Compound C significantly blocked the effects of IMD_1-53_ on restoring SOD2 activities (Figure 7G–J). Furthermore, pretreatment with Compound C blocked the effects of IMD_1-53_ on decreasing intracellular ROS and mitochondrial ROS production in calcified VSMCs, but pretreatment with LY294002 had no significant effect (Figure 7K–N).

We found that the phosphorylation of AMPK and Akt was reduced in calcified VSMCs compared to the controls, which was reversed by IMD_1-53_ treatment. The phosphorylation of PKA was not changed in calcified cells, and IMD_1-53_ treatment also had no significant effect on PKA phosphorylation (Figure 7A–D). Compound C or LY294002 preincubation blocked the inhibitory effect of IMD_1-53_ on VSMC calcification, but H89 preincubation had no significant effect (Figure 7E,F). Collectively, the inhibitory effects of IMD_1-53_ on VSMC calcification might be primarily mediated by increasing Sirt3 levels by activating the AMPK signaling pathway.

## 4. Discussion

In this study, we report the first new mechanism by which IMD attenuates VC in CKD by improving mitochondrial function and inhibiting mitochondrial oxidative stress through upregulating Sirt3. Exogenous IMD_1–53_ administration alleviated VC in CKD rats and osteogenic phenotype transformation of VSMCs. IMD_1–53_ administration also rescued the decreased MMP level and inhibited mitochondrial oxidative stress in calcified VSMCs. The mitochondrial deacetylase Sirt3 was downregulated in the calcified aortas of CKD rats and calcified VSMCs, while IMD_1-53_ treatment reversed this phenomenon. By using the SIRT3 inhibitor 3-TYP, the effect of IMD_1-53_ in attenuating VSMC calcification was eliminated. 3-TYP pretreatment also blocked IMD_1-53,_ elevating the MMP level of calcified VSMCs and subsequently inhibiting mitochondrial oxidative stress in calcified VSMCs. The use of the IMD receptor antagonist IMD_17-47_ or the AMPK inhibitor Compound C blocked the upregulation of Sirt3 by IMD_1-53_ and further blocked the effect of IMD_1-53_ on attenuating VSMC calcification by improving mitochondrial function through upregulating Sirt3. Collectively, IMD attenuates VC in CKD by improving mitochondrial function through upregulating Sirt3. IMD may upregulate Sirt3 through its receptor complex and the post-receptor signaling pathway AMPK.

The present study confirmed that IMD was a protective factor for VC under the pathological condition of CKD. Rat VC in CKD in this study was performed according to a previous study [4]. Five-six nephrectomy with vitamin D_3_ injection for 12 weeks was used to induce CKD in rats, a modeling procedure commonly used to study VC outcomes in CKD [19]. After completion of the surgery, the model needs to include vitamin D or dietary phosphorus [20,21]. Without the concurrent use of vitamin D, 5/6 nephrectomy rats develop hyperparathyroidism, which is a feature of this model. However, such model does not result in significant alterations in serum calcium levels [19]. In the CDK model induced by five-six nephrectomy with vitamin D3, a mild increase in calcium level and substantial reduction in parathyroid hormone were observed, as compared to CKD animals not receiving concurrent vitamin D_3_ [22].

Our results showed that plasma IMD level was decreased in CKD rats, in contrast, the protein levels of CRLR, RAMP2 and RAMP3 were increased in calcified aortas, which was consistent with previous findings [4]. IMD exerts its biological effects by activating the CRLR/RAMPs system [23]. However, the expression changes of IMD and its receptors vary by the state and stage of disease. The pathological environment of CKD may lead to downregulation of IMD, resulting in compensatory upregulation of the receptors.

We found that IMD attenuated VC in CKD rats by improving mitochondrial function and inhibiting mitochondrial oxidative stress. Oxidative stress is a strong contributor to the onset and development of VC [24]. Recent studies have revealed that mitochondrial oxidative stress induced by impaired mitochondrial function is an important factor contributing to VSMC calcification [25,26], therefore, inhibition of mitochondrial oxidative stress by improving mitochondrial function is considered a potential therapeutic approach to attenuate VC in CKD [8,27]. Mechanistically, osteogenic markers, such as Runx2 and BMP2, are activated in VSMCs during the development of VC [28], while oxidative stress further encourages the process [29,30]. Our previous studies found that IMD attenuated abdominal aortic aneurysm by inhibiting oxidative stress through downregulation of NADPH oxidases (NOXs) in vascular tissue [6,31]. Here, our study further extends the mechanism of the antioxidative stress effect of IMD by improving mitochondrial function and inhibiting mitochondrial oxidative stress.

Sirt3 protein levels were decreased in the calcified aortas of CKD rats and calcified VSMCs. Sirt3 is a NAD^+^-dependent deacetylase predominantly located in mitochondria [32]. Enzymatic activity of Sirt3 is reduced by mitochondrial dysfunction due to repressed mitochondrial NAD^+^ levels, leading to dysregulation of mechanisms for mitochondrial homeostasis maintenance [33,34]. Meanwhile, impaired mitochondrial function affects the utilization of acetyl-CoA in mitochondria, leading to the accumulation of acetyl-CoA in mitochondria, which impairs the function of mitochondrial proteins, such as SOD2 [35], through nonenzymatic acetylation modification [36]. The major mechanism by which Sirt3 attenuates mitochondrial ROS overproduction is to maintain low levels of acetylation of SOD2 [35]. Recently, a few studies have provided direct evidence that Sirt3 is involved in VC inhibition [12,37,38]. In our study, downregulated Sirt3, impaired mitochondrial function and elevated mitochondrial ROS levels indicated a dysregulated mechanism of Sirt3-mediated mitochondrial homeostasis maintenance in calcified VSMCs. This finding was further confirmed by elevated acetylation of SOD2 and reduced SOD2 activity.

Exogenous administration of IMD_1-53_ significantly attenuated VC in CKD and VSMC calcification, upregulated Sirt3, and improved VSMC mitochondrial function. Furthermore, the use of the Sirt3 selective inhibitor 3-TYP blocked the protective effects of IMD_1-53_ in calcified VSMCs by upregulating Sirt3, thereby reversing these phenomena. 3-TYP, an analogue of nicotinamide, is a selective inhibitor of Sirt3 [13], which is often used in Sirt3-related studies [12]. It appears that IMD may prevent VC by regulating Sirt3, and Sirt3 is required for IMD to attenuate VSMC calcification and improve mitochondrial function in calcified cells. Furthermore, IMD_1-53_ was unable to reduce VSMC calcification in Sirt3^-/-^ mice, confirming the dependence of IMD_1-53_ on Sirt3 for its protective effect on VSMC calcification. However, SIRT3 regulates the function of multiple mitochondrial proteins through deacetylation to maintain mitochondrial homeostasis [34]. Our study only reveals one specific mechanism by which IMD attenuates VC via Sirt3, and others still need further investigation.

The downstream signaling pathways of IMD receptors are PI3K/Akt, AMPK, and cAMP/PKA [3,15]. IMD regulates downstream substances through these pathways, but it differs between diseases and pathophysiological states [3,4]. In aging-associated VC, IMD_1-53_ modulates Sirt1 through three pathways [3]. In calcified VSMCs, AMPK phosphorylation was reduced, whereas IMD_1-53_ treatment restored it. AMPK is typically activated by an increase in the AMP/ATP to ATP ratio, which activates multiple downstream signaling pathways to meet cellular energy demands [39]. Due to impaired mitochondrial function, reduced ATP synthesis should activate AMPK to maintain mitochondrial homeostasis [40]. Our results suggest inactivated AMPK in calcified VSMCs, and AMPK-mediated mitochondrial homeostasis maintenance may be dysregulated. Inhibition of only the AMPK signaling pathway completely blocked the effect of IMD_1-53_ on upregulating Sirt3 in calcified VSMCs, reducing mitochondrial function and antioxidant effects mediated by Sirt3, indicating that IMD exerts its protective effects on VC through the AMPK signaling pathway. Despite the fact that the PI3K/Akt pathway is also closely related to mitochondria and energy metabolism, our results showed that inhibition of the pathway only partially prevented Sirt3 upregulation and SOD2 enzyme activity restoration induced by IMD_1-53_. In addition, PI3K/Akt inhibition did not significantly block the antioxidative effects of IMD_1-53_ in calcified VSMCs. There is a complex interaction between the PI3K/Akt and AMPK signaling pathway when cells undergo energy metabolic stress and oxidative stress [41]. PI3K/Akt and AMPK play different roles in the IMD signaling pathway for unknown reasons, but their interactions may explain the difference. In addition, AMPK and PI3K/Akt are both intracellular signaling pathways that are regulated by various factors, and PI3K/Akt might be regulated by other substances in VC. Further research is needed to understand the mechanism in detail. The detailed mechanisms of how IMD_1-53_ regulates Sirt3 require further investigation. Furthermore, future studies should be conducted using Sirt3^-/-^ mice.

Our study and others’ work [12,37] suggest that Sirt3 may be a potential target for the treatment of VC. Therefore, promoting Sirt3 activity may pave the way to overcome a barrier precluding the therapeutic use of anti-VC drugs. A large number of studies have been conducted to promote Sirt3 activity by upregulating Sirt3 expression, such as the natural products resveratrol and Honokiol [42,43]. Furthermore, by providing precursors for NAD synthesis, such as NMN, the level of the Sirt3 deacetylation substrate NAD is increased, thereby promoting the activity of Sirt3 [44]. Recently, Zhang et al. reported the screening of a series of small molecule compounds by structure-guided design and high-throughput screening and identified a compound that could specifically increase Sirt3 activity. However, this compound showed considerable lung toxicity, which might reveal this Sirt3 activator to still have certain risks for clinical applications [45]. The benefit of IMD in regulating Sirt3 is that, as an endogenous bioactive peptide, it is extensively distributed throughout the body [46].

## 5. Conclusions

In conclusion, our study discovered a novel mechanism by which IMD alleviates vascular calcification in CKD. IMD alleviated vascular calcification in CKD rats by improving mitochondrial function and inhibiting mitochondrial oxidative stress through upregulating Sirt3.

## Data Availability

Data is contained within the article and supplementary material.

## Supplementary Materials

The following supporting information can be downloaded at: https://www.mdpi.com/article/10.3390/ph15101224/s1, Figure S1: IMD and its receptor in vascular calcification of CKD rats; Figure S2: IMD treatment improved renal function of CKD rats with vascular calcification; Figure S3: Validation of primary cultured rat VSMCs and mice VSMCs; Figure S4: 3-TYP treatment increased superoxide dismutase 2 (SOD2) acetylation in rat VSMCs.
