# Supplementary material for: Intermedin Alleviates Vascular Calcification in CKD through Sirtuin 3-Mediated Inhibition of Mitochondrial Oxidative Stress
Source: Pharmaceuticals (Basel). 2022 Oct 2;15(10):1224. doi: 10.3390/ph15101224 (PMC9608591; doi:10.3390/ph15101224)
Supplement: Supplementary file 1 [file pharmaceuticals-15-01224-s001.zip › pharmaceuticals-1906500-supplementary.pdf]

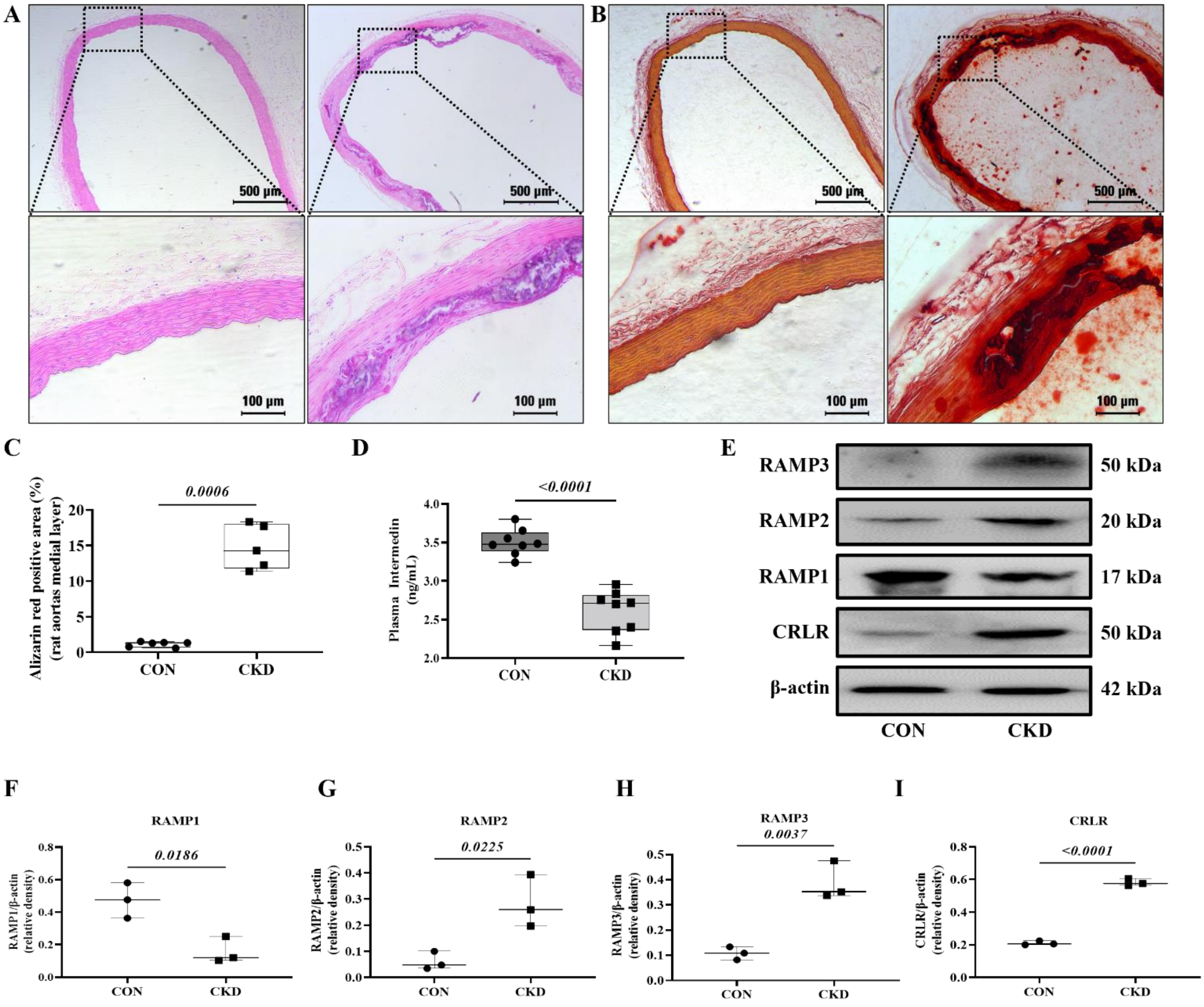

Figure S1. IMD and its receptor in vascular calcification of CKD rats. (A) Representative hematoxylin and eosin (H&E) staining (Scale bar=500 µm) and (B) Alizarin red staining for calcium deposition (positive staining: red) (Scale bar=500 µm) in rat aortas. (C) Quantification of alizarin red-positive staining (n=5-6). (D) Enzyme-linked immunosorbent assay of plasma IMD level in CKD rats (n=6). (E) Representative images of Western blot analysis of protein levels of receptor activity modifying protein (RAMP) 1, RAMP2, RAMP3 and calcitonin receptor-like receptor (CRLR) in rat aortas, and (F–I) quantification (n=3). CON (circles): control rats, CKD (squares): CKD vascular calcification rats. Data are mean ± SD.

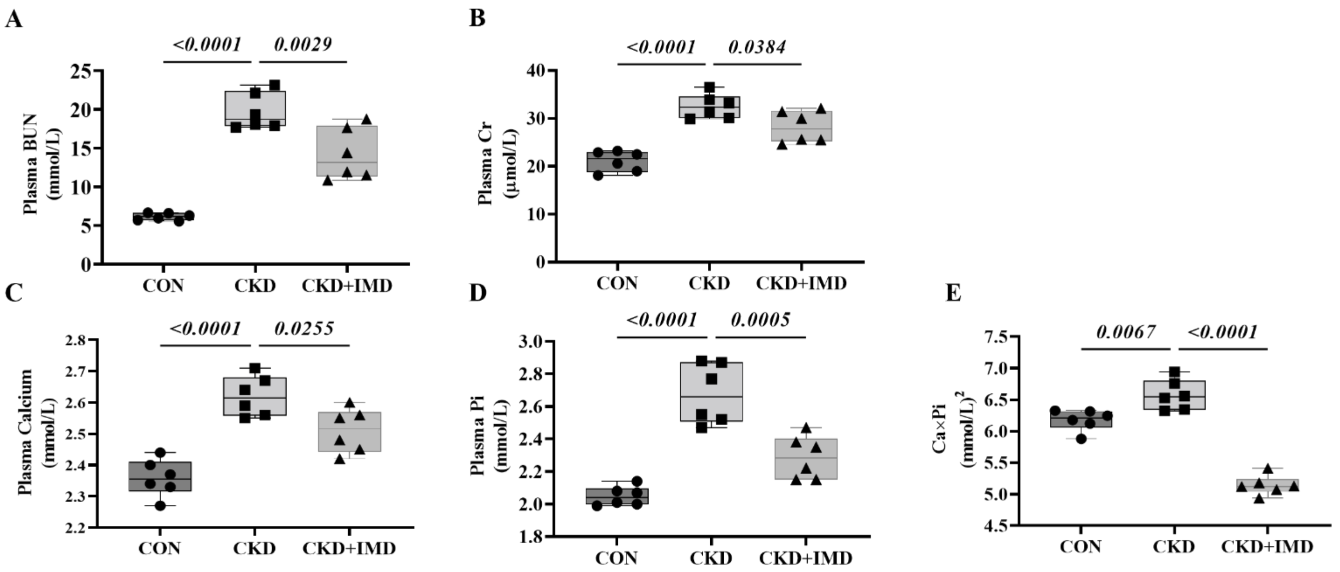

Figure S2. IMD treatment improved renal function of CKD rats with vascular calcification. Plasma biochemistry assay of (A) BUN, (B) creatinine, (C) calcium, and (D) phosphate (Pi) levels in rats with CKD (n=6). (E) Calcium phosphate product (Ca x Pi) level in rats with CKD (n=6). CON (circles): control rats, CKD (squares): CKD vascular calcification rats, CKD+IMD (triangles): CKD vascular calcification rats with IMD_{1-53} treatment. Data are mean ± SD.

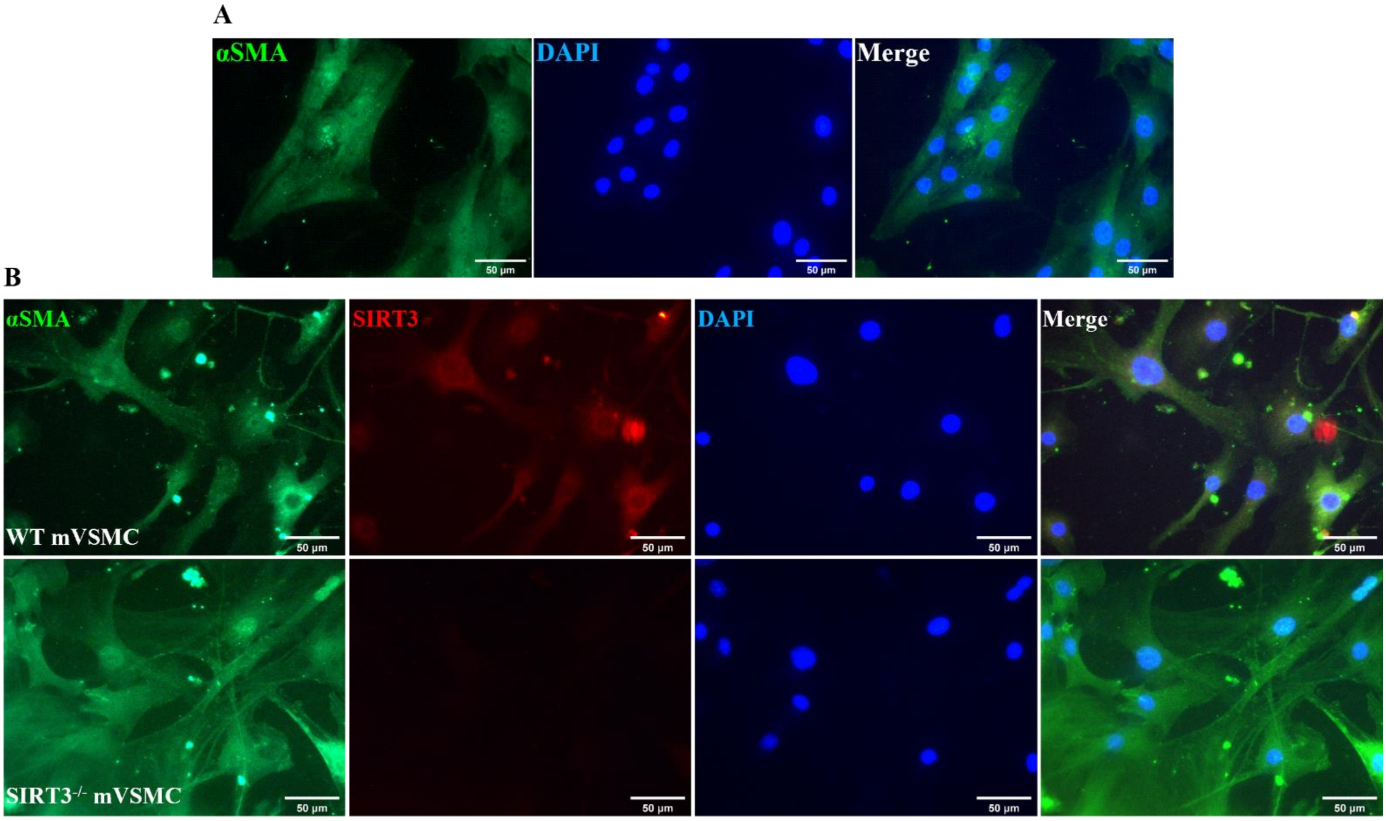

Figure S3. Validation of primary cultured rat VSMCs and mice VSMCs. (A) Representative immunofluorescence staining for alpha smooth muscle actin (αSMA) (positive: green) in primary cultured rat VSMCs. Nuclei were stained with DAPI (blue). Merged images are shown (Scale bar=50 μm). (B) Representative immunofluorescence staining for alpha smooth muscle actin (αSMA) (positive: green) and Sirtuin 3 (Sirt3) (positive: red) in primary cultured mice VSMCs. Nuclei were stained with DAPI (blue). Merged images are shown (Scale bar=50 μm).

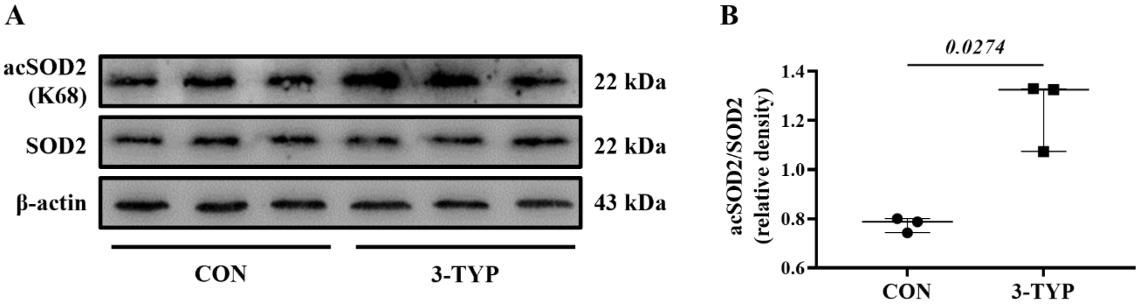

Figure S4. 3-TYP treatment increased superoxide dismutase 2 (SOD2) acetylation in rat VSMCs. (A) Images of Western blot analysis of protein levels of acetylated SOD2 (K68) and SOD2 in primary cultured rat VSMCs, and (B) quantification (n=3). CON (circles): control VSMCs, 3-TYP (squares): 3-TYP treated VSMCs. Data are mean ± SD.
